# 701 Outcomes of Patients with Burns Associated with Home Oxygen Therapy: A Systematic-Review and Meta-Analysis

**DOI:** 10.1093/jbcr/irac012.255

**Published:** 2022-03-23

**Authors:** Joshua S Yoon, Laura M Mafla, Michael Ha, Justin Kim, Patrick R Keller, Tomer Lagziel, Arthur J Nam, Panagis Galiatsatos, Charles S Hultman

**Affiliations:** R. Adams Cowley Shock Trauma Center, Division of Plastic Reconstructive and Maxillofacial Surgery, Herndon, Virginia; The Johns Hopkins University School of Medicine, Baltimore, Maryland; Division of Plastic Surgery, University of Maryland School of Medicine, Baltimore, Maryland; Division of Plastic, Reconstructive & Maxillofacial Surgery, R Adams Cowley Shock Trauma Center, Baltimore, Maryland; Department of Plastic and Reconstructive Surgery, Johns Hopkins School of Medicine, Baltimore, Maryland; Johns Hopkins University School of Medicine / Sackler School of Medicine, New-York Program, Tel-Aviv University, Rockville, Maryland; Division of Plastic, Reconstructive & Maxillofacial Surgery, R Adams Cowley Shock Trauma Center, Baltimore, Maryland; Division of Pulmonary and Critical Care Medicine, Johns Hopkins University, Baltimore, Maryland; Johns Hopkins University School of Medicine, Baltimore, Maryland

## Abstract

**Introduction:**

Home oxygen therapy (HOT) is frequently prescribed for patients with pulmonary dysfunction, which predisposes these patients to a unique health hazard at home. Given the combustibility of oxygen and method of delivery, HOT can result in burns that will affect primarily the facial area and may potentially be associated with inhalational injury. Additionally, a significant portion of patients on HOT are active smokers, which further heightens the risk of these HOT related burns. The outcomes of burn patients with burns associated with HOT is not yet completely understood. We conducted a systematic review and meta-analysis to characterize the outcomes of burn patients with burns associated with HOT.

**Methods:**

We searched MEDLINE (Pubmed) for studies that reported on outcomes of burns associated with HOT. From this search, we screened 80 articles via online platform. Through our selection criteria, 17 articles focusing on patients with HOT-related burns were selected for systematic review and meta-analysis. Data was analyzed using a data analysis program.

**Results:**

Seventeen studies observing a total of 1460 burn patients, published between 1990 and 2020, were included. Of these, one is a prospective study, twelve are retrospective chart reviews, and four were case reports/series. The mean age of the patients was 64.3 ± 4.5 years with a male to female predisposition of 1.5:1 and an average TBSA of 4.2 ± 4.1%. Of these burn patients, 93.4% were on HOT for COPD and 85.2% of injuries occurred while at home. Smoking caused 84.3% of all HOT-related burns and accrued a mean hospital charge of $73,628. With respects to clinical outcomes, the overall mortality rate was 11.3%, 33.6% required intubation, the average duration of intubation was 8.5 days, 31.3% had inhalational injury, 7.8% underwent tracheostomy, and 60.8% of patients were discharged home.

**Conclusions:**

The majority of HOT-related burns are secondary to smoking and these injuries can result in significant morbidity and mortality and associated healthcare costs. A significant portion of these injuries can be prevented with smoking cessation and/or more judicious HOT allocation, however further studies and efforts will be needed to address this issue.

